# Differential responses of the mosquito *Aedes albopictus *from the Indian Ocean region to two chikungunya isolates

**DOI:** 10.1186/1472-6785-10-8

**Published:** 2010-03-12

**Authors:** Estelle Martin, Sara Moutailler, Yoann Madec, Anna-Bella Failloux

**Affiliations:** 1 Génétique moléculaire des Bunyavirus, Institut Pasteur, 25-28 rue du Dr Roux, 75724 Paris cedex 15, France; 2 Unité de Recherche et d'Expertise Epidémiologie des Maladies Emergentes, 25-28 rue du Dr Roux, 75724 Paris cedex 15, France

## Abstract

**Background:**

*Aedes aegypti *and *Aedes albopictus *are both vectors of chikungunya virus (CHIKV). The two *Aedes *species co-exist in the Indian Ocean region and were involved in the 2005-2006 CHIKV outbreaks. In the Reunion Island, a single mutation in the viral envelope has been selected that leads to high levels of replication in *Ae. albopictus*, and a short extrinsic incubation period as the virus could be found in saliva as early as two days after infection. An important question is whether this variant is associated with adverse effects impacting some mosquito life-history traits such as survival and reproduction.

**Results:**

We performed experimental infections using three mosquito strains of *Ae. aegypti *Mayotte and *Ae. albopictus *(Mayotte and Reunion), and two CHIKV strains (E1-226A and E1-226V). *Ae. aegypti *Mayotte were similarly susceptible to both viral strains, whereas *Ae. albopictus *Mayotte and *Ae. albopictus *Reunion were more susceptible to CHIKV E1-226V than to E1-226A. In terms of life-history traits measured by examining mosquito survival and reproduction, we found that: (1) differences were observed between responses of mosquito species to the two viruses, (2) CHIKV infection only affected significantly some life-history traits of *Ae. albopictus *Reunion and not of the other two mosquito strains, and (3) CHIKV reduced the lifespan of *Ae. albopictus *Reunion and shortened the time before egg laying.

**Conclusion:**

We demonstrated that CHIKV only reduces the survival of *Ae. albopictus *from the Reunion Island. By laying eggs just before death, reproduction of *Ae. albopictus *from the Reunion Island is not reduced since other parameters characterizing oviposition and hatching were not affected.

## Background

Chikungunya virus (CHIKV) is an arbovirus (*Togaviridae *family, *Alphavirus *genus) transmitted by *Aedes *mosquito species, that usually induces an acute illness in humans characterized by fever, rash, and incapacitating arthralgia [1]. The virus was first described in Tanzania in 1952 [2]. In Africa, it has been maintained in a sylvatic cycle involving forest mosquitoes feeding preferentially on wild non-human primates [3,4]. The virus was later introduced into Asia where it circulates within an urban cycle; only an inter-human transmission cycle is described on this continent, involving *Aedes aegypti *and *Aedes albopictus *as vectors [5,6].

In early 2005, CHIKV was introduced into the Indian Ocean region, probably by viremic travelers returning from Kenya where an outbreak had started in June 2004 [7]. Later, in 2005 and 2006, CHIKV spread across the Indian Ocean islands (Comoros, Madagascar, Mayotte, Seychelles, Mauritius, the Reunion Island). Afterwards, CHIKV expanded into Asia [8-10] and Africa [11,12]. Surprisingly, an autochthonous CHIKV outbreak was reported in Europe in the Ravenna region of Italy during the summer of 2007 [13]. Most recent CHIKV outbreaks were related to the mosquito *Ae. albopictus *whose geographical distribution has been expanding into new tropical and temperate regions over the past decades [14]. In the Indian Ocean region, *Ae. aegypti *and *Ae. albopictus *are present together or alone; both vectors can be involved in the CHIKV transmission cycle.

Phylogenetic analyses based on sequences of the E1 gene encoding an envelope glycoprotein revealed that the Indian Ocean isolates belonged to the Eastern/Central/Southern African genotype [15]. A switch to a new variant operated in the Reunion Island from the end of 2005, where most CHIKV isolated from patients presented an amino-acid substitution in the E1 glycoprotein, from an alanine (E1-226A) to a valine (E1-226V). This mutation enhances transmission by *Ae. albopictus *[16]. The new variant spread easily through the Indian Ocean region and has thus been found in countries where *Ae. albopictus *is the main *Aedes *species. Subsequent CHIKV outbreaks were related to the transmission of the E1-226V variant by *Ae. albopictus*.

By experimental infections, we demonstrated that from day 3 post-infection (pi), *Aedes *mosquitoes could produce 1,000-fold more viral particles than those ingested [17]. Although it was believed for many years that infection with an arbovirus had no deleterious effects upon its arthropod host, recent studies indicated that such viral infections may not be benign. When compared with uninfected individuals, viral infections in mosquitoes can reduce fecundity [18], the ability to get a blood-meal [18,19], and survival [20-22]. The effect on the survival of the mosquito vector is important in determining vector capacity. How a vector responds to infection by a virus? Among the strategies developed to compensate for the cost of infection by a pathogen, a host could reduce the harm caused by infection by tolerance, which involves the alteration of host life-history traits. These latter are defined as traits relevant to the allocation of resources in reproduction, survival and growth: for example the number of offspring, age of first reproduction, adult body size etc.

In this work, we have analyzed the effects of CHIKV infection on survival and reproduction of two mosquito species, *Ae. aegypti *and *Ae. albopictus*. Changes in survival and reproduction induced upon infection with two CHIKV strains, the original E1-226A strain and the new E1-226V variant were studied.

## Results

### Susceptibility of *Aedes *species to CHIKV strains

**Figure 1:**
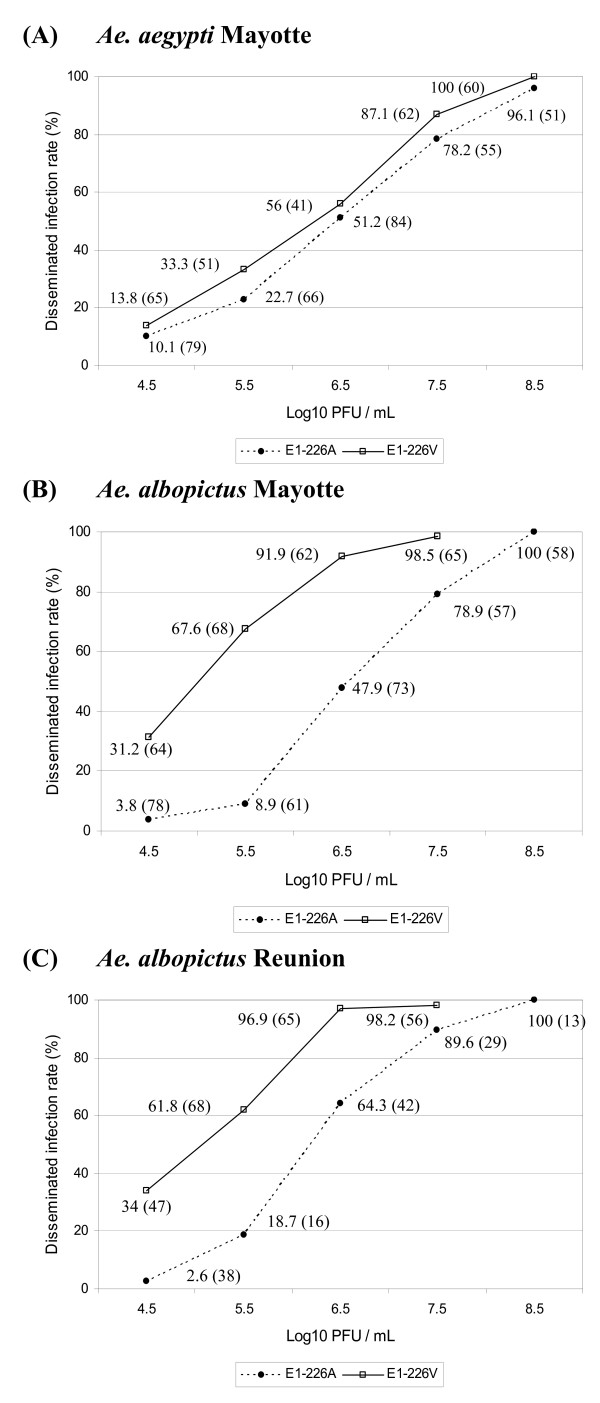
**Disseminated infection rates estimated at different viral titers**. (A) *Ae. aegypti *Mayotte, (B) *Ae. albopictus *Mayotte, (C) *Ae. albopictus *Reunion. Rates were estimated 14 days after exposure to infectious blood-meals by IFA on head squashes. At each time point, the disseminated infection rates are given, and in brackets the number of mosquitoes tested.

Batches of mosquitoes were orally exposed to serial 10-fold dilutions of CHIKV E1-226A and CHIKV E1-226V (Figure 1). To determine whether disseminated infection rates correlated with blood-meal titers, surviving mosquitoes were analyzed at day 14 pi by IFA on head squashes. *Ae. aegypti *Mayotte showed similar susceptibility towards both CHIKV E1-226A and CHIKV E1-226V (p > 0.05); at a titer of 10^6.5 ^PFU/mL, 56.1% of mosquitoes showed a disseminated infection when infected with CHIKV E1-226V and 51.2% did with respect to CHIKV E1-226A (Figure 1A). On the other hand, *Ae. albopictus *Mayotte were found to be 1-2 fold more susceptible to CHIKV E1-226V than to CHIKV E1-226A (p < 0.01); at a titer of 10^6.5 ^PFU/mL, 47.9% of mosquitoes presented a disseminated infection when infected with CHIKV E1-226A, in contrast to 91.91% with CHIKV E1-226V (Figure 1B). Lastly, *Ae. albopictus *Reunion displayed the same profile as *Ae. albopictus *Mayotte, with significant differences between viruses at each titer point (p < 0.01) except when the titer was high (e.g. 10^7.5 ^PFU/mL) where both viral strains gave similar disseminated infection rates (p = 1). All *Aedes *species examined presented a similar susceptibility to CHIKV E1-226A at a titer of 10^6.5 ^PFU/mL (p > 0.05). At this same viral titer, *Ae. albopictus *(Mayotte and Reunion) presented a higher susceptibility to CHIKV E1-226V than did *Ae. aegypti *Mayotte (Figure 1C). The plateau corresponding to 100% of disseminated infection rate was reached for blood-meal titers higher than 10^8.5 ^PFU/mL.

### Survival of mosquitoes

**Figure 2:**
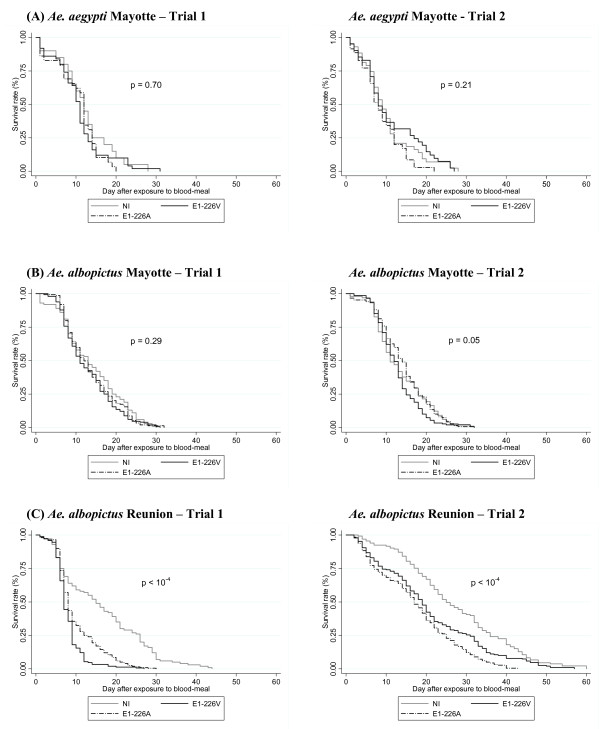
**Kaplan-Meier estimates for probability of survival after blood-meal**. (A) *Ae. aegypti *Mayotte, (B) *Ae. albopictus *Mayotte, (C) *Ae. albopictus *Reunion. Mosquitoes were fed on a non-infectious blood-meal or an infectious blood-meal at a titer of 10^7.5 ^PFU/mL.

The survival rates for each mosquito species, depending on the trial and according to the virus (NI, CHIKV E1-226A, CHIKV E1-226V), are presented in Figure 2. The infectious blood-meals were at a titer of 10^7.5 ^PFU/mL.

For *Ae. aegypti *Mayotte (Figure 2A), each trial led to the same results: the survival curves were not significantly different according to the infection (p = 0.70 and p = 0.21 for trials 1 and 2, respectively). A parametric model was used to estimate the effects on survival of the strain of the virus (NI, CHIKV E1-226A, CHIKV E1-226V) and of the infection status of the mosquito (NI, NDINF, DINF), and to assess the existence of an interaction between these two factors. For this purpose, we combined trials 1 and 2 as no significant difference was detected in NI mosquitoes (p = 0.24), or mosquitoes exposed to CHIKV E1-226A (p = 0.23) or to CHIKV E1-226V (p = 0.94). The risk of death was higher for NDINF mosquitoes compared to NI mosquitoes (Time ratio [TR] (95% confidence interval) [CI]: 0.60 (0.42 - 0.85) with CHIKV E1-226V and 0.49 (0.33 - 0.72) with CHIKV E1-226A). On the other hand, no significant difference in the risk of death was observed between DINF mosquitoes and NI mosquitoes infected with CHIKV E1-226V (TR (95% CI): 1.19 (0.91 - 1.56) and with CHIKV E1-226A (1.10 (0.81 - 1.48)) (Table 1).

**Table 1:** Life duration of *Aedes *mosquitoes

Viral strain	Status	Life duration ± SD (N)					
		
		*Ae. aegypti *Mayotte		*Ae. albopictus *Mayotte		*Ae. albopictus *Reunion	
		
		1	2	1	2	1	2
	NI	12 ± 7 (20)	10 ± 7 (43)	14 ± 8 (100)	14 ± 7 (98)	16 ± 11 (100)	27 ± 13 (133)

E1-226A	NDINF	9.5 ± 7 (8)	5 ± 3 (12)	9 ± 4 (18)	13 ± 8 (38)	8 ± 5 (31)	14 ± 11 (20)
	DINF	11 ± 5 (21)	11 ± 5 (23)	14 ± 6 (131)	15 ± 6 (111)	11 ± 5 (169)	18 ± 10 (160)

E1-226V	NDINF	7 ± 8 (14)	9 ± 5 (12)	13 ± 6 (29)	13 ± 5 (49)	7 ± 5 (36)	21 ± 18 (19)
	DINF	12 ± 5 (36)	12 ± 8 (29)	13 ± 7 (119)	13 ± 5 (99)	8 ± 3 (113)	21 ± 12 (170)

The table shows the life duration of *Ae. aegypti *and *Ae. albopictus *from Mayotte and the Reunion Island after infection with two CHIKV strains: CHIKV E1-226A and CHIKV E1-226V. N corresponds to the number of female analysed, SD to the standard deviation, 1 and 2 to respectively, trial 1 and trial 2, DINF to females with disseminated infection, NDINF to females with non-disseminated infection, and NI to non-infected females.

For *Ae. albopictus *Mayotte (Figure 2B), the two trials mainly led to the same results: survival curves did not differ significantly according to the virus (p = 0.29 and p = 0.05 for trials 1 and 2, respectively). To compare the survival curves, we combined the two trials since no significant difference was found between NI mosquitoes (p = 0.53), mosquitoes exposed to CHIKV E1-226A (p = 0.55) and CHIKV E1-226V (p = 0.55). Using a parametric survival model, compared to NI mosquitoes, mosquitoes exposed to CHIKV E1-226V were not at greater risk of dying, whether the infection status was NDINF (TR (95% CI): 1.01 (0.87-1.18)) or DINF (TR (95% CI): 1.01 (0.91-1.13)). On the other hand, for mosquitoes exposed to CHIKV E1-226A, NDINF mosquitoes showed a higher risk of dying than did NI mosquitoes (TR (95% CI): 0.80 (0.67-0.95)) while DINF mosquitoes had a significantly lower risk of dying (TR (95% CI): 1.15 (1.03-1.28)) than did NI mosquitoes (Table 1).

For *Ae. albopictus *Reunion (Figure 2C), the two trials led to the same results: a significant difference in the survival curves was detected according to the virus (both p < 10^-4^). Mosquitoes exposed to CHIKV E1-226A or CHIKV E1-226V had lower survival rates than NI mosquitoes. As the trials differed significantly when NI mosquitoes (p < 10^-4^), those exposed to CHIKV E1-226A (p < 10^-4^) or to CHIKV E1-226V (p < 10^-4^) were analyzed, trials were considered separately. Using a parametric survival model, we found that for mosquitoes exposed to CHIKV E1-226V, NDINF mosquitoes, as well as DINF mosquitoes, were more at risk of dying than NI mosquitoes in both trials (for NDINF mosquitoes, TR (95% CI): 0.43 (0.35-0.54) and 0.52 (0.37-0.74) in trials 1 and 2, respectively; for DINF mosquitoes: 0.63 (0.54-0.74) and 0.70 (0.59-0.83) in trials 1 and 2, respectively). For mosquitoes exposed to CHIKV E1-226A, NDINF and DINF mosquitoes were more at risk of death than NI mosquitoes were, in both trials: for NDINF mosquitoes, 0.46 (0.37-0.58) and 0.39 (0.28-0.54) in trials 1 and 2, respectively; and for DINF mosquitoes: 0.74 (0.65-0.86) and 0.62 (0.52-0.73) in trials 1 and 2, respectively (Table 1).

### Oviposition characteristics

#### Time to first egg laying

In *Ae. aegypti *Mayotte, the Kaplan-Meier estimates showed no significant difference in the time to egg laying according to the CHIKV strain (p = 0.20) or the infection status of the mosquito (p = 0.73). Combining CHIKV strain and infection status as a single factor, the parametric survival model showed no effect of these two factors on the time to egg laying (p = 0.47). In *Ae. albopictus *Mayotte, the Kaplan-Meier estimates showed no significant difference in the time to egg laying when the virus (p = 0.25) or the infection status of the mosquito (p = 0.31) were examined. The parametric survival model did not show any effect of the virus and infection status of mosquito on the time to egg laying (p = 0.50). In contrast, in *Ae. albopictus *Reunion, the Kaplan-Meier estimates showed significant differences in the time to egg laying when considering the virus (p = 0.03) and the infection status of mosquito (p = 0.003). The parametric survival model showed a significant effect of the virus and infection status of mosquitoes on the time to egg laying (p < 10^-4^). Mosquitoes exposed to CHIKV E1-226V and CHIKV E1-226A laid eggs significantly earlier than NI mosquitoes (Table 2).

**Table 2:** Oviposition characteristics (delay to laying, number of laid eggs and time between first oviposition and female death)

Viral strain	Status	Delay to laying ± SD (N)						Number of laid eggs ± SD (N)						Time between first oviposition and female death ± SD (N)					
		
		*Ae. aegypti *Mayotte		*Ae. albopictus *Mayotte		*Ae. albopictus *Reunion		*Ae. aegypti *Mayotte		*Ae. albopictus *Mayotte		*Ae. albopictus *Reunion		*Ae. aegypti *Mayotte		*Ae. albopictus *Mayotte		*Ae. albopictus *Reunion	
		
		1	2	1	2	1	2	1	2	1	2	1	2	1	2	1	2	1	2
	NI	6 ± 1 (10)	6 ± 2 (22)	7 ± 3 (73)	6 ± 2 (80)	10 ± 5 (24)	14 ± 8 (83)	50 ± 18 (10)	39 ± 27 (22)	33 ± 23 (73)	35 ± 23 (80)	30 ± 32 (24)	56 ± 43 (83)	9 ± 8 (20)	7 ± 6 (43)	9 ± 7 (100)	8 ± 7 (98)	14 ± 12 (100)	19 ± 14 (133)

E1-226A	NDINF	7 ±3 (2)	5 ± 0 (4)	5 ± 0 (16)	7 ± 3 (22)	8 ± 3 (11)	9 ± 7 (13)	42 ± 46 (2)	53 ± 39 (4)	35 ± 19 (16)	34 ± 20 (22)	50 ± 49 (11)	41 ± 38 (13)	8 ± 7 (8)	3 ± 3 (12)	5 ± 5 (18)	9 ± 8 (38)	8 ± 6 (31)	8 ± 8 (20)
																			
	DINF	6 ± 2 (7)	6 ± 2 (14)	6 ± 2 (96)	7 ± 3 (82)	9 ± 5 (62)	12 ± 7 (86)	45 ± 18 (7)	37 ± 26 (14)	34 ± 26 (96)	30 ± 22 (82)	40 ± 35 (62)	41 ± 41 (86)	9 ± 5 (21)	7 ± 5 (23)	10 ± 7 (131)	9 ± 7 (111)	7 ± 7 (169)	11 ± 11 (160)

E1-226V	NDINF	5 ± 2 (6)	5 ± 1 (3)	8 ± 4 (24)	7 ± 3 (39)	7 ± 4 (9)	9 ± 8 (8)	35 ± 15 (6)	41 ± 28 (3)	35 ± 27 (24)	24 ± 19 (39)	32 ± 41 (9)	72 ± 58 (8)	5 ± 7 (14)	8 ± 4 (12)	7 ± 5 (29)	7 ± 6 (49)	5 ± 5 (36)	17 ± 19 (19)
																			
	DINF	7 ± 2 (26)	6 ± 1 (17)	7 ± 3 (91)	7 ± 3 (74)	8 ± 3 (43)	12 ± 9 (89)	54 ± 17 (26)	52 ± 31 (17)	32 ± 24 (91)	32 ± 23 (74)	38 ± 35 (43)	49 ± 43 (89)	7 ± 7 (36)	9 ± 6 (29)	8 ± 7 (119)	8 ± 6 (99)	5 ± 4 (113)	14 ± 14 (170)

*Ae. aegypti *and *Ae. albopictus *from Mayotte and the Reunion Island were infected with two CHIKV strains: CHIKV E1-226A and CHIKV E1-226V. N corresponds to the number of female analysed, SD to the standard deviation, 1 and 2 to respectively, trial 1 and trial 2, DINF to females with disseminated infection, NDINF to females with non-disseminated infection, and NI to non-infected females.

#### Number of eggs laid per mosquito

The number of eggs laid according to the mosquito species, virus and infection status of the mosquito is described in Table 2. Using a negative binomial regression model for each mosquito species, we did not find any evidence that the number of eggs laid differed according to the virus and infection status of the mosquito (data not shown: p = 0.79, 0.82, and 0.47 in *Ae. aegypti *Mayotte, *Ae. albopictus *Mayotte and *Ae. albopictus *Reunion, respectively).

#### Time between first oviposition and mosquito death

We also described the time between the first oviposition and mosquito death using Kaplan-Meier survival curves performed separately for each mosquito species. No difference according to the virus and infection status of the mosquito was observed in *Ae. aegypti *Mayotte (p = 0.33) and *Ae. albopictus *Mayotte (p = 0.14). In *Ae. albopictus *Reunion, both trials led to the same conclusion: when compared to NI mosquitoes, the time was significantly shorter in mosquitoes infected with CHIKV E1-226A or CHIKV E1-226V irrespective of whether the infection status of the mosquito was NDINF or DINF (p = 0.007 and p = 0.001 for trials 1 and 2, respectively).

### Hatching characteristics

#### Proportion of mosquitoes with at least one egg hatched

When examining the proportion of females with at least one egg hatched, no significant differences were found whatever the mosquito species, the virus and the infection status of the mosquito, when results were considered for *Ae. aegypti *Mayotte (p = 0.71) and *Ae*. *albopictus *Mayotte (p = 0.85). In contrast, for *Ae. albopictus *Reunion a significant difference was found to be attribuable to NI mosquitoes (p = 0.02) (Table 3).

**Table 3:** Hatching characteristics (proportion of females with at least one egg hatched and hatching rate per female)

Viral strain	Status	Proportion of females with at least one egg hatched (%) (N)								Hatching rate per female ± SD (N)			
		
		*Ae. aegypti* Mayotte		*Ae. albopictus* Mayotte		*Ae. albopictus* Reunion		*Ae. aegypti* Mayotte		*Ae. albopictus* Mayotte		*Ae. albopictus* Reunion	
		
		1	2	1	2	1	2	1	2	1	2	1	2
	NI	70 (10)	50 (22)	52 (86)	51 (73)	50 (24)	80 (79)	23.9 ± 25.7 (10)	29 ± 32.4 (22)	8.4 ± 13.1 (73)	9.5 ± 14.6 (80)	15.2 ± 22.2 (24)	36.1 ± 31.0 (83)

E1-226A	NDINF	100 (2)	50 (4)	50 (6)	38 (16)	55 (11)	69 (13)	44.1 ± 46.6 (2)	36.6 ± 43.5 (4)	6.6 ± 10 (16)	12.9 ± 15.5 (22)	23.2 ± 31.7 (11)	29.1 ± 32.5 (13)
	DINF	57 (7)	71 (14)	45 (76)	45 (96)	45 (62)	63 (65)	21.1 ± 22.4 (7)	32.7 ± 31.1 (14)	9.9 ± 17.7 (96)	13.5 ± 15.5 (82)	17.3 ± 26.3 (62)	32.6 ± 33.6 (86)

E1-226V	NDINF	33 (6)	100 (3)	42 (19)	54 (24)	33 (9)	75 (8)	8.5 ± 16.2 (6)	51.3 ± 35.7 (3)	9.5 ± 12.1 (24)	19.8 ± 22.5 (39)	12.0 ± 23.6 (9)	35.2 ± 37.1 (8)
	DINF	31 (26)	76 (17)	44 (73)	51 (91)	65 (43)	73 (81)	5.9 ± 13.0 (26)	33.5 ± 29.7 (17)	10.9 ± 16.2 (91)	19.6 ± 20.2 (74)	30.1 ± 28.7 (43)	40.1 ± 34.0 (89)

The table refers to the hatching characteristics of *Ae. aegypti *and *Ae. albopictus *from Mayotte and the Reunion Island infected with two CHIKV strains: CHIKV E1-226A and CHIKV E1-226V. N corresponds to the number of female analysed, SD to the standard deviation, 1 and 2 to respectively, trial 1 and trial 2, DINF to females with disseminated infection, NDINF to females with non-disseminated infection, and NI to non-infected females.

#### Hatching rate per mosquito

When the hatching rate per mosquito was examined, *Ae. aegypti *Mayotte mosquitoes were found to show a slight difference according to the viral strains (p = 0.03) and not to the infection status of the mosquito (p = 0.15). Similarly, *Ae. albopictus *Mayotte mosquitoes only showed a significant difference when viral strains were considered (p < 10^-4^) and not the infection status of the mosquito (p = 0.94). Finally, *Ae. albopictus *Reunion also showed differences related to viral strains (p < 10^-2^) and not to the infection status of the mosquito (p = 0.12). In most cases, mosquitoes infected with CHIKV E1-226V displayed the highest hatching rates (Table 3). Eggs that issued from infected or non-infected mosquitoes maintained in a BSL-3 laboratory usually showed a lower capacity to hatch than eggs that were kept in an insectarium under standard conditions (data not shown).

### Replication of CHIKV in mosquitoes

**Figure 3:**
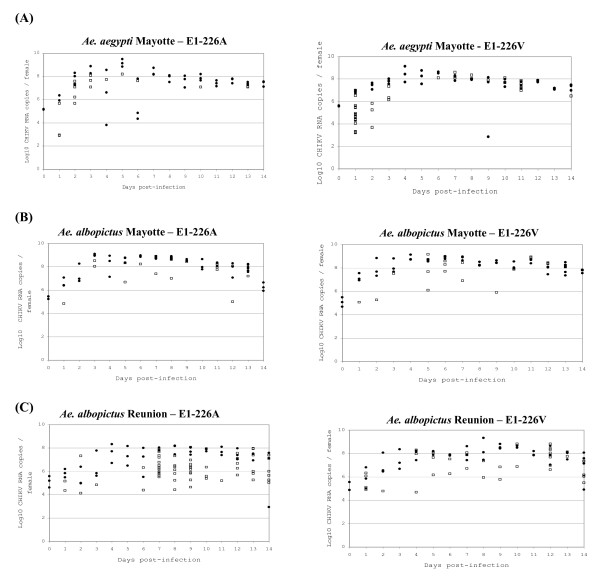
**Quantitative analysis of replication in mosquitoes**. (A) *Ae. aegypti *Mayotte, (B) *Ae. albopictus *Mayotte, and (C) *Ae. albopictus *Reunion were exposed to blood-meals with CHIKV E1-226A and CHIKV E1-226V at a titer of 10^7.5 ^PFU/mL. Dead ('white square') and live('black circle') mosquitoes were examined.

Dead mosquitoes were collected once a day. The number of viral RNA copies present in dead mosquitoes did not vary within a period of 24 h after mosquito death (data not shown). For *Ae. aegypti *Mayotte, mosquitoes had an average of 10^5.1 ^(± 10^0.01^) viral RNA copies per mosquito when a blood-meal containing CHIKV E1-226A was ingested, and an average of 10^5.6 ^(± 10^0.02^) viral RNA copies per mosquito that had ingested CHIKV E1-226V (Figure 3A). Following the blood-meal, viral load increased to reach a maximum of 10^9.1 ^± 10^0.3 ^viral RNA copies at day 5 pi for CHIKV E1-226A and between day 4 (10^8.4 ^± 10^0.7^) and day 6 pi (10^8.6 ^± 10^0.1^) for CHIKV E1-226V. Later, a slight decrease was observed until day 14 pi (10^7.4 ^± 10^0.2 ^for CHIKV E1-226A and 10^7.3 ^± 10^0.3 ^for CHIKV E1-226V). For both viruses, many mosquitoes died between day 1 and day 3 pi, especially at day 1 for *Ae. aegypti *Mayotte infected with CHIKV E1-226V.

For *Ae. albopictus *Mayotte, mosquitoes ingested an average of 10^5.4 ^(± 10^0.1^) viral RNA copies when exposed to a blood-meal containing CHIKV E1-226A and 10^5.1 ^(± 10^0.4^) with CHIKV E1-226V (Figure 3B). The maximum viral load was reached at day 3 pi after ingestion of both CHIKV E1-226A (10^9.0 ^± 10^0.1^) and CHIKV E1-226V (10^8.1 ^± 10^0.6^) followed by a plateau until day 11 pi. At day 14 pi, the number of viral RNA copies had decreased to 10^6.5 ^(± 10^0.8^) for CHIKV E1-226A and 10^7.7 ^(± 10^0.1^) for CHIKV E1-226V.

In the case of *Ae. albopictus *Reunion, mosquitoes ingested an average of 10^5.1 ^(± 10^0.5^) RNA copies in the blood-meal containing CHIKV E1-226A and 10^5.1 ^(± 10^0.1^) RNA copies for CHIKV E1-226V (Figure 3C). After a slight increase in the amount of viral RNAs from day 1 to day 3 pi, a plateau was observed until day 13 pi: 10^7.3 ^± 10^0.3 ^with CHIKV E1-226A and 10^7.9 ^± 10^0.3 ^with CHIKV E1-226V. Dead females exhibited two distinct profiles: (i) females which ensured a viral replication at a level similar to that of live females and (ii) females which did not ensure any viral replication. This is more clear-cut for ALPROV infected with CHIKV E1-226A.

## Discussion

Whichever viral strain was examined (E1-226A or E1-226V), differential responses to CHIKV infection were found according to the mosquito species (*Ae. aegypti versus Ae. albopictus*) and their geographical origin (Mayotte *versus *the Reunion Island). CHIKV infection affected the life-history traits of *Ae. albopictus *from the Reunion Island. We have found that apart from the viral strains, CHIKV infection induced effects on survival, by reducing lifespan, and on oviposition characteristics, by shortening the time to egg laying of *Ae. albopictus *from the Reunion Island.

*Aedes albopictus *is a mosquito species native to Southeast Asia that has spread beyond its natural habitat by means of intercontinental trades [23]. This mosquito is able to transmit various arboviruses in laboratory conditions [14]. In addition to this species, *Ae. aegypti *is native to Africa and comprises two forms: the forest-dwelling *Ae. a. formosus *involved in the enzootic transmission of arboviruses, and the domestic form *Ae. a. aegypti *whose distribution has extended over the tropical region acting as the main vector of dengue viruses [24,25]. Both species are container-breeding mosquitoes that share the same breeding sites. In Asia, the introduced *Ae. aegypti *tends to displace *Ae. albopictus *from urban settlements [26,27], whereas the spread of *Ae. albopictus *has been associated with declines of *Ae. aegypti *in the Americas [28,29]. In the Indian Ocean region, *Ae. albopictus *was introduced several centuries ago [30] and in the Reunion Island, it was first recorded in 1913 [31]. On the island, this mosquito became the most common *Aedes *species [32] since the control program against malaria vectors in the 1950s that led to the confinement of *Ae. aegypti *in remote areas [33]. On Mayotte, *Ae. aegypti *was first recorded in 1943 [34] and *Ae. albopictus *was observed later, in 2001 [35]. Larvae of both species could be found in artificial containers making inter-specific resource competition possible [36,37]. *Ae. albopictus *predominates in domestic habitats in Mayotte [38].

The CHIKV outbreak began in Kenya in 2004, and subsequently spread to the islands of the Indian Ocean [39]. A new strain of CHIKV emerged that carried a point mutation, which changes the amino acid at position 226 in the E1 envelope glycoprotein from an alanine to a valine [15]. This single mutation has increased the vector competence of *Ae. albopictus *for CHIKV [16,40]. This increased transmissibility of CHIKV is one of the hypotheses evoked to explain the expansion of CHIKV transmission into new regions such as Madagascar [41] and Cameroon [11] in 2006, and India [8], Italy [13] and Gabon [12] in 2007. Prior to these recent CHIKV outbreaks, *Ae. aegypti *had been considered the primary epidemic vector of CHIKV and *Ae. albopictus*, a secondary vector. A single E1-A226V mutation was sufficient to increase the ability of CHIKV to infect *Ae. albopictus *modifying the virus infectivity for a vector species other than the typical *Ae. aegypti *vector. Although the mutation confers a selective advantage in *Ae. albopictus*, there was no corresponding advantage in *Ae. aegypti*. Our results showed that *Ae. aegypti *Mayotte was similarly susceptible to both viral strains, whereas *Ae. albopictus *Mayotte and *Ae. albopictus *Reunion were more susceptible to the CHIKV E1-226V than to the CHIKV E1-226A strain (Figure 1). This may explain the predominance of E1-226V in countries where *Ae. albopictus *is considered to be the main vector species. At a titer of 10^7.5 ^PFU/mL, E1-226V virus was disseminated more effectively by *Ae. albopictus *(Mayotte and Reunion) than by *Ae. aegypti *(Mayotte). Infection of *Ae. aegypti *Mayotte requires that the CHIKV blood-meal titers are significantly higher than for infection of *Ae. albopictus *(Mayotte and Reunion), which suggests that once introduced, E1-226V would be positively selected by *Ae. albopictus*.

As an intracellular parasite, CHIKV can mediate changes in host life-history traits. Two sorts of mechanisms could be proposed. On one hand, the use of host resources to maintain viral replication can lead to modifications of resource allocation as pathogenic effects. On the other hand, life-history modifications may be responses of the host to counterbalance the negative effects of parasitism. By reacting to the parasite, the host reduces the effect of infection on its fitness [42]. We have shown that replication of CHIKV in *Aedes *mosquitoes is intensive, e.g. 1,000 more viral particles in females from day 3 pi, and saliva became infectious from two days pi [17]. In the present work, we have analyzed the effects of CHIKV replication on several life-history traits of *Aedes *mosquitoes related to resource allocation, and only found significant modifications in *Ae. albopictus *from the Reunion Island. Thus CHIKV infection has a significant effect on the time until death and egg laying. Moreover, such negative effects were independent of the viral strain: CHIKV E1-226A and CHIKV E1-226V displayed similar effects. Nevertheless, aside from CHIKV infection,*Ae. albopictus *from the Reunion Island better survived in BSL-3 laboratory conditions than the two other mosquito strains. Regardless, infection by CHIKV has a negative impact on the survival of *Ae. albopictus *from the Reunion Island (Figure 2C). Lifespan was shortened by 6-9 days when mosquitoes were infected with CHIKV E1-226A or CHIKV E1-226V. Moreover, by shortening the time from blood-feeding to laying, CHIKV-infected *Ae. albopictus *from the Reunion Island allocates an increased reproduction investment to laying eggs before death. Thus only limited consequences may affect vector dynamics, as other factors dictating oviposition (the number of eggs laid per mosquito) and hatching (the proportion of females with at least one egg hatched and hatching rate) characteristics were only slightly altered by infection. Such earlier reproduction has been reported in other animal-parasite systems [43,44]. Infection by a pathogen may modify resource distribution: infected hosts will allocate more resources to reproduction, subtracting them from those attributed to growth and survival [45,46]. Nevertheless, the question remains as to why this phenomenon is not species-specific. Effectively, *Ae. albopictus *from Mayotte did not exhibit the same pattern. One explanation could be that *Ae. albopictus *assumed to be introduced into the Reunion Island by immigrants from Asia in the 17^th^-18^th ^centuries [47] has been subjected to major bottlenecks that cause strong genetic divergence from the Asian mosquitoes [48]. Thus *Ae. albopictus *from the Reunion Island may be genetically different from *Ae. albopictus *detected in Mayotte in 2001 [35].

From our results, a very provocative scenario could be envisioned concerning CHIKV evolution during the Indian Ocean outbreak. Since Mayotte is located halfway between Kenya, where the outbreak began, and the Reunion Island, where the CHIKV E1-A226V was first reported, this new variant could have already been present in Mayotte. In Mayotte, no data pertaining to CHIKV sequences was available before 2006 and CHIKV E1-226V was only identified after this time [15]. In Mayotte, both vectors co-exist [35] and we clearly showed that *Ae. aegypti *Mayotte was similarly susceptible to both viral strains while *Ae. albopictus *Mayotte was more susceptible to the CHIKV E1-226V strain. The mutation could therefore be interpreted as a positive selection phenomenon for better transmission by *Ae. albopictus*. In addition, we found that infection with CHIKV E1-226A or CHIKV E1-226V did not induce significant consequences on either species from Mayotte. Nevertheless, on the Reunion Island, the unique vector *Ae. albopictus*, maintains a better transmission of CHIKV E1-226V compared to CHIKV E1-226A. However, an important decrease in the lifespan of *Ae. albopictus *from the Reunion Island was reported as a consequence of infection followed by changes in mosquito reproduction. We propose that CHIKV E1-226A was maintained on Mayotte by both *Ae. aegypti *and *Ae. albopictus*. Later, the introduction of CHIKV E1-226A onto the Reunion Island where *Ae. aegypti *is rare, would have led to the selection of the new variant by *Ae. albopictus*. Since then, CHIKV E1-226V has invaded some neighboring islands where *Ae. albopictus *is present. Thus, CHIKV E1-226V probably reached Mayotte later, after having been selected in the Reunion Island. In addition, the role of other mutations in the viral genome in the adaptation of CHIKV to *Aedes *mosquitoes could not be completely excluded [49]. Furthermore, some characteristics of the *Ae. albopictus *bio-ecology might have favored CHIKV transmission in urban areas: high anthropophily and high population densities.

## Conclusion

Our results are in agreement with modifications in life-history traits reported for parasitized animals. We found that the species *Ae. albopictus *was more susceptible to the CHIKV E1-226V than to the CHIKV E1-226A strain which assumes that once introduced, E1-226V would be positively selected by *Ae. albopictus*. Nevertheless, CHIKV infection only induced negative impacts on survival and on egg hatching characteristics of *Ae. albopictus *from the Reunion Island which led us to suggest that *Ae. albopictus *from the Reunion Island might be less adapted to CHIKV than mosquitoes from Mayotte. However, the physiological mechanisms underlying this pattern are unknown. Nevertheless, the outcome of the pathogen/host interaction often involves the immune system that is restricted to the innate immune function in insects. This is an effective mechanism to provide protection against a wide variety of pathogens including viruses. We have evaluated the number of viral RNA copies in mosquitoes (live females sacrificed every day and also dead females; see Figure 3) and found that females that died a few days after infection did not host more viral particles than did live females. Thus we might suggest that mosquitoes did not die following an excess of viral replication but more probably when mounting an immune response. Modifications of life-history traits and/or mounting an efficient antiviral response should be among strategies developed by the vector to optimize its fitness.

## Methods

### Mosquitoes

Mosquitoes were from two islands in the Indian Ocean: (i) Mayotte (Petite-Terre) where *Ae. aegypti *Mayotte and *Ae. albopictus *Mayotte were collected in December 2006 and (ii) the Reunion Island (Providence) where *Ae. albopictus *Reunion was collected in March 2007. In Mayotte, *Ae. albopictus *was detected in 2000 [35] and this is now the most frequent *Aedes *species encountered in urban and suburban habitats on this island [38]. On the Reunion Island, *Ae. albopictus *predominates in urban settings, while *Ae. aegypti *is present as residual non anthropophilic populations [33]. Experiments were carried out with the 3^rd^, 6^th ^and 2^nd ^laboratory raised generations for *Ae. aegypti *Mayotte, *Ae. albopictus *Mayotte, and *Ae. albopictus *Reunion, respectively. Mosquitoes were maintained in insectariums at 28 ± 1°C with 80% relative humidity and a 16 h:8 h photoperiod. Batches of eggs were put to hatch and pools of 200 larvae were reared in pans containing 1 liter of water supplemented with 1-2 tablets of cat food. Only females were used for oral infections. This rearing procedure was used in order to obtain females of similar size, making them likely to take equal quantities of blood; this was checked using a procedure for measuring blood-meal size [50]. The females obtained were assumed to ingest a similar number of viral particles [51,52]. Adults were fed with a 10% sucrose solution until infection.

### CHIKV strains

Two CHIKV strains were isolated from human sera of patients on the Reunion Island: strain E1-226A was isolated in June 2005 and strain E1-226V in November 2005; these two strains differed mainly by a substitution of alanine by valine at position 226 of the E1 glycoprotein, in a region of the protein that is predicted to interact with the target membrane [15]. The two CHIKV strains were provided by the French National Reference Center for Arboviruses at the Institut Pasteur, which had obtained the verbal consent from the patients who provided blood sera. Stocks of both viral strains were produced on *Ae. albopictus *C6/36 cells that were infected at a multiplicity of infection (moi) of 0.1 PFU (plaque forming units)/cell for 48h at 27°C. Supernatant fluids were collected and viral titers estimated by serial 10-fold dilutions on Vero cells. Each virus stock was divided into aliquots and stored at -80°C until use.

### Oral infections of mosquitoes

For each mosquito species, batches of 60 one-week-old females were isolated in plastic boxes and starved for 24 hours before infection. Then they were allowed to feed for 15 min through a chicken skin membrane, which covered the base of a feeder that contained the infectious meal maintained at 37°C. The blood-meal was composed of a virus suspension diluted (1:3) in washed rabbit erythrocytes with adenosine triphosphate (5 × 10^3 ^M).

### Susceptibility of *Aedes *species to CHIKV strains

To determine the susceptibility of *Ae. aegypti *Mayotte and *Ae. albopictus *(Mayotte and Reunion) to CHIKV E1-226A and CHIKV E1-226V, we exposed batches of mosquitoes to a 10-fold dilution of viral stocks ranging from 10^4.5 ^to 10^8.5 ^PFU/mL. Only fully engorged mosquitoes were transferred to small cardboard containers and maintained at 28°C for 14 days. At the end of this period when optimal dissemination of the virus is obtained [16], mosquitoes that had survived were sacrificed and tested for the presence of CHIKV by immunofluorescence assay (IFA) on head squashes [53]. To this end, the head was severed from the body, and sets of 10-15 heads were placed on one slide. A second slide was placed on this to squash the heads. Slides were then immersed in acetone for 20 min at -20°C. Head squashes were incubated for 30 min at 37°C with a first anti-CHIKV antibody prepared from a mouse ascite diluted in PBS 1× (1:200). After three washes in PBS 1×, the squashes were incubated with a goat anti-mouse conjugate diluted in PBS 1× (1:80) supplemented with Evans blue. Slides were observed under an epifluorescence microscope. In replication-negative mosquitoes, the infection is limited to the midgut and head tissues appear in red. In replication-positive mosquitoes, the virus spreads beyond the midgut and infects secondary organs including the salivary glands. In such mosquitoes, the head tissues appear in green. A significant correlation has been found between the presence of virus in the head and the ability of a female mosquito to excrete virus through saliva [17]. Dissemination of the virus could be achieved within two days. The disseminated infection rate corresponds to the number of females with disseminated infection (mosquitoes with head tissues in green) among surviving females 14 days after an infectious meal.

### Mosquito life-history traits

To determine whether infection by CHIKV has an impact on mosquito life-history traits, other batches of mosquitoes of each mosquito species were allowed to feed on three different blood-meals: two infectious blood-meals containing CHIKV E1-226A or CHIKV E1-226V, and one non-infectious blood-meal that used Dulbecco's modified Eagle's medium instead of the virus suspension. Females selected at this stage had been inseminated by males after emergence in cages. The titer of the infectious blood-meal was 10^7.5 ^PFU/mL [16]. Fully engorged mosquitoes were individually isolated in 50 mL plastic tubes where a piece of wet cotton was placed at the bottom as support for oviposition. Mosquitoes maintained at 28°C were fed with a 10% sucrose solution until death. For each combination of CHIKV strain and mosquito species, the experiment was conducted twice; the number of mosquitoes considered in each experiment was variable depending on the number of engorged mosquitoes obtained.

For each mosquito, three traits were examined: survival, oviposition and egg hatching. Survival was evaluated by scoring the number of dead mosquitoes every day, to estimate mosquito lifespan. The infection status of each mosquito was determined at the date of death by IFA on head squashes: (i) a head found to be positive by IFA corresponded to a mosquito with a disseminated infection (DINF) - after crossing the midgut, the virus had disseminated inside the hemocele, colonizing different organs including the central nervous system; (ii) a negative head according to IFA corresponded to a mosquito with a non-disseminated infection (NDINF) - the virus did not cross the midgut and was retained in the epithelium cells. All mosquitoes have ingested infectious viral particles as ~10^5 ^PFU have been detected in engorged females sacrified one hour after ingestion of the infectious blood-meal (data not shown). Non-infected mosquitoes named (NI) had ingested a non-infectious blood-meal. Oviposition was examined according to three parameters: (i) the time to first laying after the blood-meal, which allows the duration of the gonotrophic cycle of each mosquito to be determined, (ii) the total number of eggs laid per mosquito, (iii) the time between the first oviposition and mosquito death.

Eggs were stored at 28°C until hatching. Hatching was described by assessing two parameters: (i) the proportion of mosquitoes among those which have laid eggs that have at least one egg hatched and (ii) the hatching rate, which corresponds to the proportion of eggs hatched among those laid per mosquito. Eggs were put to hatch by immersion in dechlorinated tap water in an insectarium maintained at 28°C.

### Quantitative RT-PCR

To determine whether mosquito mortality was related to an over- replication of CHIKV, three living mosquitoes, and dead mosquitoes if present, were collected every day. For each mosquito, the total RNA was extracted using a nucleospin RNA II kit (Macherey-Nagel) according to the manufacturer's instruction [16]. To construct the standard curve, a synthetic CHIKV RNA transcript was generated. A PCR product containing the target region was prepared using CHIKV, and this was cloned into the pCR II TOPO vector (Invitrogen). The product amplified using vector-specific primers was purified using a PCR Purification Kit (Qiagen). RNA transcripts were produced *in vitro *using the RiboMAX™ Large Scale RNA Production System (Promega) appropriate for either SP6 or T7 RNA polymerase. The transcript size was 1,356 bp for both CHIKV E1-226A and CHIKV E1-226V. Residual DNA was eliminated by several DNAse treatments (Turbo DNA-free (Ambion)). After quantification using a spectrophotometer, the RNA transcript solution was stored at -80°C.

One-step quantitative RT-PCR was performed in a volume of 25 μl containing 3 μl RNA template, 12.5 μl 2 × Brilliant SYBR Green I QPCR Master Mix (Stratagene), 1 μl sense (2.5 μM) and 1 μl anti-sense (2.5 μM) primers, 0.25 μl Fluorescein (1 μM), and 0.0625 μl Stratascript RT/RNAse block enzyme. Primers were selected in the E2 structural protein regions: sense Chik/E2/9018/+ (CACCGCCGCAACTACCG) and anti-sense Chik/E2/9235/- (GATTGGTGACCGCGGCA). The amplification program in a i-Cycler TM (Biorad) included a reverse transcription step at 50°C for 30 min, an inactivation of RT/RNAse enzyme step at 95°C for 10 min followed by 40 cycles of 95°C 30 s, 56°C 1 min, 72°C 30 s, a step at 95°C for 1 min, and 81 cycles of 55°C (+0.5°C/cycle) 30 s. The size of the amplification product was 217 bp. PCR was performed in triplicate for each mosquito. Signals were normalized to the standard curve obtained using serial dilutions of synthetic RNA transcript. Normalized data were used to measure the number of RNA copies in infected mosquitoes according to ΔCt analysis. One Log of PFU corresponds to 1-2 Log RNA virus (unpublished data).

### Statistical analysis

For each mosquito species, disseminated infection rates were compared according to CHIKV and viral titers using a χ^2 ^test, the Fisher's exact test being used in the case of small sample sizes.

The survival rate for each trial and each mosquito species according to the virus (NI, CHIKV E1-226A and CHIKV E1-226V) were described using Kaplan-Meier survival curves. These survival curves were then compared using the logrank test. For each mosquito species, the effects on survival of a 5-category covariate that combines virus and infection status (NI, NDINF, DINF) was investigated, after adjustment to the trial, using an accelerated failure time model assuming that the survival time was log-normally distributed. Time ratios (95% confidence intervals) were estimated and tested using Wald's parametric test. This model was chosen as the proportional assumption model required by the more classical Cox model would not have been valid.

For each mosquito species infected (or non-infected) by a given virus, the time to the first egg laying was also described using Kaplan-Meier estimates, and curves were compared using the logrank test. We also chronicled the time between the first oviposition and mosquito death, using Kaplan-Meier estimates, and compared these curves using the logrank test. Then, the effect on the total number of eggs laid of the covariance that combined virus and infection status (NI, NDINF, DINF) was investigated using a negative binomial regression model. This model is relevant when analyzing incidence as it enables the control according to lifespan, and it provides incidence rate ratios and their 95% confidence intervals. The significance level of the covariate was tested using Wald's test.

For each mosquito species, the hatching capacity was studied through an assessment of the proportion of mosquitoes with at least one hatched egg. The proportions of mosquitoes in each of the five categories defined by virus and infection status were compared using a Fisher's exact test. Hatching rates, i.e. the proportion of hatched eggs among all eggs laid by a mosquito, were compared using analysis of variance, according to virus and infection status.

All statistical analyses were performed using the STATA software (StataCorp LP, Texas, USA).

## Authors' contributions

EM performed the research. SM helped to design the study. YM analyzed the data and helped to draft the manuscript. ABF conceived the study, analyzed the data, and wrote the manuscript. All authors read and approved the final version of the manuscript.
